# Alzheimer's Disease (AD): Mini‐Mental State Exam (MMSE) Scores Before and After COVID‐19 Pandemic

**DOI:** 10.1002/alz70857_103447

**Published:** 2025-12-25

**Authors:** Caroline Komatsu, Ricardo S. Komatsu

**Affiliations:** ^1^ Scientific Police, Sao Paulo, Sao Paulo, Brazil; ^2^ FAMEMA ‐ Marilia Medical School, Marilia, Sao Paulo, Brazil

## Abstract

**Background:**

Neuropsychiatric symptoms (NPS) are core features of AD and COVID‐19 pandemic had a strong effect in people with AD, affecting overall outcomes and decreasing quality of life.

**Method:**

It is an applied *ex‐post‐facto* and data survey research, with a quantitative approach.

**Results:**

The median educational level of this sample of 91 elderly with AD (median age: 77 years old) was 3,9 years. Before COVID‐19 pandemic, the median MMSE score was 19,25. After COVID‐19 pandemic, the median MMSE score was 19.

**Conclusion:**

In this sample of patients with AD, COVID‐19 pandemic did not significantly influence the MMSE scores, indicating that more research is demanded to examine the pandemic effects over time.

